# Iron Metabolism in Cancer Progression

**DOI:** 10.3390/ijms21062257

**Published:** 2020-03-24

**Authors:** Stefania Forciniti, Luana Greco, Fabio Grizzi, Alberto Malesci, Luigi Laghi

**Affiliations:** 1 Humanitas Clinical and Research Center, IRCCS, Department of Gastroenterology—Laboratory of Molecular Gastroenterology, Rozzano, 20089 Milan, Italy; stefania.forciniti@humanitasresearch.it (S.F.); luana.greco@humanitasresearch.it (L.G.); 2 Department of Immunology and Inflammation, Humanitas Clinical and Research Center, Via Manzoni 56, Rozzano, 20089 Milan, Italy; fabio.grizzi@humanitasresearch.it; 3 Humanitas Clinical and Research Center, IRCCS, Department of Gastroenterology, Rozzano, 20089 Milan, Italy; alberto.malesci@hunimed.eu; 4 Department of Medicine and Surgery, University of Parma, 43100 Parma, Italy

**Keywords:** iron, *HFE* (gene), oxidative stress, macrophages

## Abstract

Iron is indispensable for cell metabolism of both normal and cancer cells. In the latter, several disruptions of its metabolism occur at the steps of tumor initiation, progression and metastasis. Noticeably, cancer cells require a large amount of iron, and exhibit a strong dependence on it for their proliferation. Numerous iron metabolism-related proteins and signaling pathways are altered by iron in malignancies, displaying the pivotal role of iron in cancer. Iron homeostasis is regulated at several levels, from absorption by enterocytes to recycling by macrophages and storage in hepatocytes. Mutations in *HFE* gene alter iron homeostasis leading to hereditary hemochromatosis and to an increased cancer risk because the accumulation of iron induces oxidative DNA damage and free radical activity. Additionally, the iron capability to modulate immune responses is pivotal in cancer progression. Macrophages show an iron release phenotype and potentially deliver iron to cancer cells, resulting in tumor promotion. Overall, alterations in iron metabolism are among the metabolic and immunological hallmarks of cancer, and further studies are required to dissect how perturbations of this element relate to tumor development and progression.

## 1. Introduction

**Figure 1:**
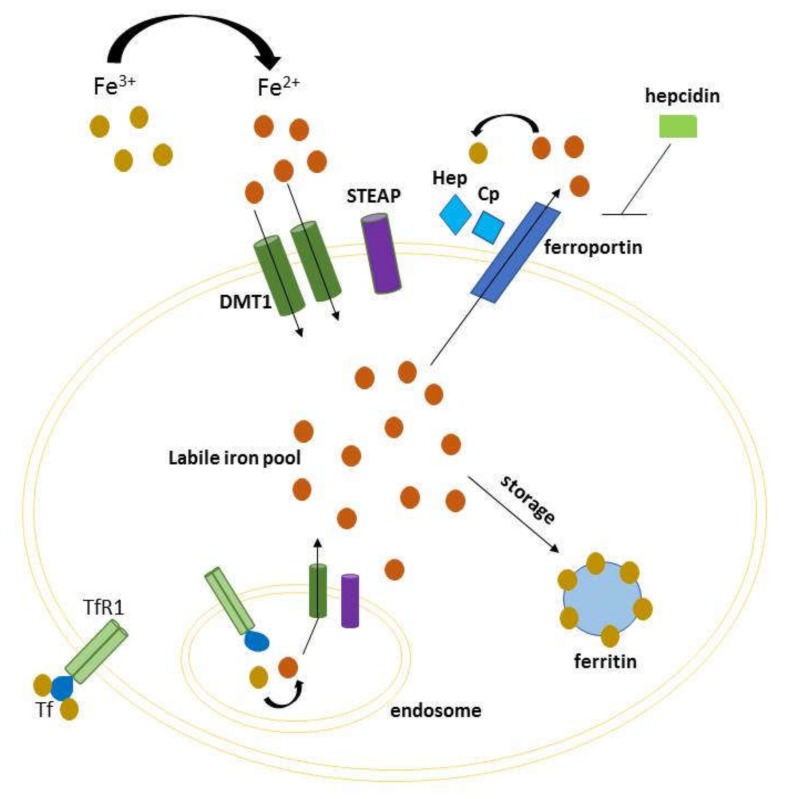
Schematic representation of cellular iron homeostasis. Iron is imported via the divalent metal-ion transporter 1 (DMT1) transporter (requiring STEAP2 activity), or via endocytosis of transferrin receptor 1. Intracellular iron can either constitute the cytoplasmic labile iron pool (LIP, to be delivered to different cell compartments), either be stored in ferritin for later use. The excess of iron is exported through ferroportin, which cooperates with hephaestin and ceruloplasmin, and inhibited by hepcidin.

Iron is an indispensable element for several cellular processes, as many basic functions of the cell rely upon iron. It is chiefly involved in processes such as DNA synthesis, ATP production and oxygen transport. In humans, iron deposits are distributed in macrophages, reticulocytes, liver, and the equilibrium between its absorption and export is controlled in the intestine [1]. Multiple molecules cooperate to maintain iron homeostasis because, if the equilibrium is disrupted, iron can catalyze Fenton reaction and produce reactive oxygen species (ROS) [2]. This event may promote ferroptosis, a non-apoptotic cell death mechanism characterized by iron-dependent generation of lipid peroxidation products [3,4]. Iron homeostasis is thus a finely tuned process, both at the systemic and cellular level, which involves incorporation, utilization and storage. Iron binds to transferrin (TF) forming a complex that recognizes the transferrin receptor 1 (TFR-1) on the outer side of cell membrane. This complex is internalized in the endosome by endocytosis, and Fe^3+^ is reduced to Fe^2+^ by iron reductase, mainly by members of six-transmembrane epithelial antigen of the prostate (STEP1-4) family [5]. Thereafter, iron is transported in the cytosol mainly via divalent metal-ion transporter 1 (DMT1) by constituting the cytoplasmic labile iron pool (LIP). This now metabolically active iron can be delivered to different cell compartments for a variety of metabolic needs, or otherwise stored in ferritin, a protein complex that concentrates iron in an inactive form for later use [6]. An excess of iron can be exported out of the cell through ferroportin, the only known cellular iron efflux pomp which cooperates with ceruloplasmin or hephaestin to maintain cellular iron homeostasis [7]. In this respect, the homeostasis at cellular level is achieved by the system of iron responsive elements–iron regulatory proteins (IREs–IRPs) [8], while at systemic level is maintained by hepcidin, a circulating hormone that regulates iron levels causing degradation of ferroportin (Figure 1) [9]. An increasing number of studies have highlighted the link between dysregulation of iron metabolism and human diseases, including cancer [10,11,12]. Various iron-related proteins have been related to cancer progression and metastasis, having an altered expression and activity in cancer cells compared with the normal counterpart [13]. Particularly, iron-regulated genes increase their metabolic iron demands needed for both early and late stages of tumor development. In cancer patients, perturbation of iron metabolism contributes to anemia as seen in chronic diseases, due to several factors, among others the secretion of inflammatory factors that reduce erythropoiesis [14]. The involvement of iron in cancer is also described in mice models [15] in which a low-iron diet before inoculating cancer cells delayed tumor growth. Furthermore, it has been shown that tumors can utilize the natural iron deposits in spleen and liver. Consequently, red blood cells recycling and iron storage is compromised, and erythropoiesis reduced. In this review, we provide an overview of iron metabolism, genetic iron overload disorders, and iron-driven molecular mechanisms with respect to cancer progression. In addition, we discuss how the regulation of immune system and iron metabolism are strictly related, so that changes in either one of the two can affect the other.

## 2. Historical Bases

### 2.1. Relationship Between Iron Metabolism and Cancer

Deregulated iron homeostasis is one of the metabolic hallmarks of malignant cancer cells, in which iron is highly required during all stages of tumor development, survival, proliferation, and metastasis [16]. Being tumor cells strongly dependent on iron for their growth/proliferation, they are more sensitive to iron depletion than normal cells [17]. The pathways of iron metabolism that are deregulated in cancer include processes of uptake-export, storage, and regulation [18]. Generally, transferrin receptor1 (TFR 1) is important for iron uptake and for the regulation of cell growth. It has been demonstrated that cancer cells show a high expression of this receptor compared to normal cells [19]. For this reason, TFR1 affects cancer cell proliferation, migration, invasion, and metastasis and it represents an important target for cancer therapy [20]. 

Cui et al. found a higher expression of TFR1 in well differentiated colorectal cancers with no lymph node involvement and no distant metastasis than in normal tissues. Furthermore, patients with TFR1-positive expression had increased survival than patients with TFR1-negative expression. Although it has been detected an overexpression of TFR1 in colorectal cancer, the authors demonstrated that a downregulation of TFR1 promoted cell migration and invasion via JAK/STAT pathway [21].

TFR1 is also highly expressed in pancreatic ductal adenocarcinoma. Jeong et al. reported that TFR1 contributed to the mitochondrial respiration and ROS production in human pancreatic cancer cells which are essential in their tumorigenic growth and survival. Moreover, TFR1 expression determined the sensitivity of cells to oxidative stress [22].

It is known that iron reductase, in particular members of STEAP family, are important for iron uptake and iron reduction in the endosome [23]. Several studies demonstrate an altered expression of STEAP 1–4 in different types of tumor such as prostate, breast, pancreatic cancer and glioma [24,25]. In prostate cancer, STEAP2 seems to drive cancer progression by promoting proliferation, migration and invasion and modifying the transcriptional profiles of some genes involved in metastatic process. In this context, Burnell and colleagues demonstrated that decreasing STEAP 2 expression in prostate cancer cells reduces their proliferation, migration and invasion in vitro. Furthermore, analyzing the expression of STEAP 2 in 164 tumor tissues, it has been found an increased expression in tumor tissues compared to normal tissues [26]. A central role in iron storage is carried out by ferritin. In some cases of cancer patients, ferritin is detected in high concentration in plasma, correlating with advanced tumor stage and poor outcome [27,28]. Accordingly, increased ferritin levels are found not only in inflammatory diseases, but also in several cancers (e.g., lung carcinoma, pancreatic, and colorectal cancer), suggesting that high ferritin might underlie cancer development and progression [29,30,31].

The iron efflux is controlled by ferroportin, an iron efflux pump, and by its regulator hepcidin, which appear to be involved in tumorigenesis [32]. In prostate and breast cancer, a reduction of ferroportin expression on cell surface induces an increase of intracellular iron, and is associated with the emergence of an aggressive phenotype [33,34].

Pinnix and colleagues demonstrated that both ferroportin and hepcidin are normally expressed in human breast epithelial cells and tissues. Moreover, the ferroportin expression is strongly reduced in breast cancer cells and tissues compared to normal counterparts, while the overexpression of ferroportin reduced growth of xenografted breast cancer cells in vivo.

Furthermore, the gene expression profiles of around 800 breast cancer patients have reported that a decrease in ferroportin gene expression is correlated with an important reduction in metastasis-free and disease-free survival [35]. Numerous studies have shown that hepcidin contributes to cancer proliferation and progression, then regulating its level to reduce iron pool in tumor cells may be a new strategy in cancer treatment [36].

In colorectal cancer, an increase in intracellular iron is coupled with both an increased expression of import proteins (CYTB, DMT1, and TFR1), and a block in iron export due to aberrant localization of FPN, the loss of which was also associated with more advanced disease [37]. In colorectal cancer cells (CaCo-2 and SW480), iron overload cells caused cellular proliferation and E-cadherin repression, suggesting transition towards a mesenchymal states (epithelial to mesenchymal transition, EMT) and loss of adherence. In support of the potential role of iron-regulating proteins in EMT, it should be noted that B2-microglobulin supports metastasis in vivo by interacting with *HFE*, and its expression in cancer cells drives EMT (decreasing E-cadherin while increasing *N*-cadherin and vimentin). Accordingly, inhibition of both B2-microglobulin and *HFE* reverts EMT [38]. This aspect should be elucidated in those cancers for which an association has been found with both *HFE* genotypes (see below) and EMT [39].

### 2.2. Hereditary Hemochromatosis, a Genetic Iron-Overload Disorder, and Its Association with Cancer

Iron homeostasis is often altered by mutations that occur in several genes and that contribute to most iron-related genetic diseases resulting in iron overload or deficiency [40]. Hereditary iron overload, known as hereditary hemochromatosis (HH), is mostly associated with *HFE* gene mutations, although other genes may be less frequently involved, alike those encoding hepcidin or transferrin receptor 2 [41]. *HFE* is a MHC class-like protein that controls the expression of hepcidin, a peptide secreted in plasma by the liver that regulates iron distribution at systemic level [42]. *HFE* interacts with TFR1 to form a complex that is disrupted by binding holotransferrin. Subsequently, *HFE* cooperates with TFR2 and with the bone morphogenetic protein (Bmp) co-receptor Hemojuvelin, the interaction of which leads to the activation of hepcidin transcription [43]. Mutations in the *HFE* gene lead to an inability of hepatocytes to detect increased levels of iron and to stimulate hepcidin transcription. Consequently, this results in low hepcidin levels, high ferroportin activity, and excessive absorption of iron from diet resulting in iron overload in liver and other organs [44]. The most common mutations in the *HFE* gene implicated in the pathogenesis of HH are p.C282Y and p.H63D. C282Y is due to a substitution of tyrosine for cysteine at 282 position in the protein sequence, while H63D is a point mutation that changes histidine to aspartic acid at 63 position [45]. Most patients are homozygous for C282Y mutation, that is the predominant genetic profile in HH, or compound heterozygous for C282Y in association with H63D, the latter alone being considered a genetic profile that only predisposes to a mild iron overload. Genetic mutations of H63D in homozygosity represent approximately 1% of individuals with gene mutation [46]. It has been widely reported that *HFE* gene mutations are correlated with increased cancer risk [47], the underlying mechanism being that the accumulation of iron induces oxidative DNA damage and free radical activity. The iron-catalyzed free radical reactions cause lipid peroxidation, protein modification and DNA damage. In particular, the H63D polymorphism is associated with an increased risk of several tumors, including hepatocellular carcinoma, breast, colorectal, and pancreatic cancers [48,49]. Beside an inherent increased risk (19-fold) of developing hepatocellular carcinoma [48], carriers of *HFE* C282Y homozygotes have twice the risk of colorectal (HR 2.28; 95%C.I., 1.22–4.25) and breast cancer compared with individuals without the C282Y variant [50]. Such an increased risk was not detected in these studies for heterozygous *HFE* variants yet [51], the risk of colorectal cancer might be increased by compound heterozygosity for the *HFE* mutations (O.R.,3.03; 95% C.I., 1.06–8.61), although this association could not remain significant after Bonferroni correction for two post hoc tests. Meta-analyses focusing on the issue of the association between cancer and *HFE* variants, reported conflicting results. In some meta-analysis, C282Y but not H63D variant was related to elevated cancer risk [52], including colorectal cancer [53] under recessive model (OR = 2.00, 95 % CI = 1.32–3.04), in Caucasians. The latter association was evident for studies concerning more than 500 cases.

However, others have found that also H63D variant could be associated with increased cancer risk, such as gastric cancer (non-cardia, OR = 1.60, CI = 1.16–2.21, and intestinal subtype, OR = 1.82, CI = 1.27–2.62) [54]. A similar association has been observed also for pancreatic cancer [55], as *HFE* rs1799945 (i.e., H63D) was significantly associated with PC risk, with each additional copy of minor allele T being associated with a 1.21-fold increased risk of PC (OR = 1.21, 95 % CI = 1.05–1.39, *P* = 7.72 × 10^−3^) in Chinese population including 1000 cases and as many controls. Previously, a European study comprising 168 patients with pancreatic adenocarcinoma had failed to detect any significant association with *HFE* genotype [56]. The linkage between H63D polymorphism and the development of cancer was verified by examining published statistical data through a meta-analysis. This study estimated that H63D variant influences cancer progression, due to altered iron metabolism [57]. Specifically, it has been identified a statistically significant association between H63D polymorphism and hepatocellular carcinoma and pancreatic cancer risk, especially in Asian and African populations [57].

Further studies are needed to clarify the association between *HFE* genotypes and high risk of gastrointestinal cancers and, whether confirmed, to unravel the correlation with natural history and clinical behavior. As a companion to meta-analysis, in silico data exploration would be of help. This approach has not been used for the above mentioned gastrointestinal cancers, but it has employed to assess the frequency of *HFE* variants in glioblastoma [58] by comparing the datasets in the TCGA with those of the 1000 genome. Although no differences in survival were reported in this study, it would be of interest to extend such an approach to other cancers, possibly taking into account relevant molecular tumor features. In the case of colorectal cancer, it would be relevant to assess the frequency of *HFE* genotypes primarily as to tumor MS-status, due to the implications of this molecular phenotype for diagnosis and patient management [59]. Only one report exists as to the association of these polymorphisms as potential modifiers of Lynch syndrome, which reported an increased risk of developing colorectal cancer for H63D homozygotes [60].

### 2.3. Mini-Summary

Available studies indicate that abnormalities in iron homeostasis are among the hallmark of cancer, being traceable at tumor development, progression and metastasis. The main deregulation of iron homeostasis occurs at the level of its uptake-export or storage, thus resulting in overload or deficiency. Furthermore, variants in the *HFE* gene lead to the accumulation of iron in various organs, sustaining the pathogenesis of hereditary hemochromatosis, coupled to an increased cancer risk due to oxidative damage of DNA and free radical activity, adding these mechanisms to carcinogenesis. Further studies are warranted to better understand the correlation between *HFE* genotypes and high risk of cancer.

## 3. Molecular Mechanisms

### 3.1. The Role of Iron in Oxidative Stress

Iron is a central element for cell survival and many biological processes, thus its appropriate amount is indispensable for cells. However, when iron is present in excess it can generate reactive oxygen species (ROS), both directly and indirectly [61]. The redox-active iron (Fe^2+^) participates with hydrogen peroxide (H_2_O_2_) in the Fenton reaction (Fe^2+^+H_2_O_2_→ Fe^3+^+•OH+OH^−^) directly generating hydroxyl radical and ferric iron (Fe^3+^). This reaction catalyzed by iron produces a large amount of highly reactive hydroxyl radicals that can damage DNA, proteins and lipids through oxidation [62]. Iron associated with DNA induces mutations or single and double strand breaks, while iron bound to proteins can promote a H_2_O_2_-dependent redox signaling. Furthermore, iron is essential in the formation of lipid peroxyl radicals and it can react with lipid peroxides produced by hydroxyl radicals. These lipid peroxyl radicals can become cytotoxic aldehydes that can react with DNA damaging it [63]. The activity of several iron-dependent enzymes can indirectly generate a large amount of ROS, such as superoxide radicals and peroxides. Cytochrome P450 enzymes, nitric oxide synthases, NADPH oxidases and lipoxygenases are the most important enzymes involved in ROS generation [64,65]. In normal conditions the antioxidant enzymes, such as glutathione peroxidase, superoxide dismutase and catalases, intervene in detoxifying these ROS [66]. Obviously, the process of ROS generation and detoxification must be strictly balanced to avoid oxidative stress and consequent damage to the iron-containing proteins such as ferritin, DNA damage and in general cellular stress. It is known that ROS accumulation activates different signaling pathways that lead to cell death, such as the apoptosis signal-regulating kinase 1 (ASK1)-p38/c-Jun N terminal kinase (JNK) pathway. Since iron is involved in ROS production, it contributes to cell death pathways including apoptosis and necrosis [67]. An important finding was the identification of the ferroptosis, a mechanism of regulated cell death associated with excessive iron levels, and thus production of reactive free radicals, mainly characterized by cytological changes such as decreased mitochondria crista and a damaged outer mitochondrial membrane. For instance, iron attached to membrane phospholipids catalyzes the beginning of lipid peroxidation reactions, which have been correlated with ferroptotic cell death. A crucial regulator of ferroptosis is the glutathione peroxidase 4 (GPX4), which neutralizes lipids derived from cellular metabolism, thus protecting the cells. The direct or indirect inhibition of GPX4 can cause ferroptosis that promotes the antitumor activity of some tumor suppressors (p53, PTEN, pRb) [68]. Even if cancer cells undergo a high oxidative stress maintaining a balanced level of iron, ferroptosis does not often happen in the cancer development. It is known that the activation of Ras-mitogen-activated protein kinase (MEK) signaling can contribute to the sensitivity of cancer cells to ferroptosis, through the abundant presence of iron in cancer resulting from the regulated expression levels of transferrin receptor and ferritin [69]. The ROS- and iron-overload cell death became a new target of therapeutic strategy in several disease, including cancer. Indeed, it has been shown that some ferroptosis-inducing agents, such as erastin or sorafenib, have anti-tumor activity [68].

### 3.2. Hemeoxygenase-1 Induction in Cancer Progression

The electrophilic stimuli, oxidative stress, cellular injury and disease induce the activity of heme-oxigenase 1 (HO-1), a metabolizing enzyme that degrades the pro-oxidant heme into biliverdin/bilirubin, ferrous iron, and carbon monoxide. HO-1 is expressed at basal level in the spleen and liver but it may be induced in other organs following various stimuli (ROS, ipoxia, heat). HO-1 expression is regulated mainly at transcriptional level through the activity of Nrf2, NF-κB, and various oncogenes that can be activated in conditions of oxidative stress. The physiological roles of HO-1 are iron recycling and maintenance of homeostasis but it is also involved in pathological conditions [70]. It has been demonstrated that, the overexpression of HO-1 is correlated with a worsening of disease states, as in some types of tumor in which HO-1 is highly expressed by correlating with poor prognosis. In these cases, HO-1 acts as survival molecule and its up-regulation protects cells from apoptosis, induces cell proliferation, metastasis, angiogenesis by promoting vascular endothelial growth factor (VEGF) expression, and resistance to therapy [71]. Numerous papers describe the involvement of HO-1 in the progression of blood malignancies and in the mechanisms leading to chemo-resistance in leukemia, but also in the progression of solid tumors, such as human renal cell carcinoma, melanoma, and prostate- and pancreatic cancer [72]. Particularly, different studies show that HO-1 is expressed in cytoplasm and also in nucleus of cancer cells in lung, prostate and oral tumor tissues. In addition, the positive HO-1 immunoreactivity was detected in stromal compartment, mainly in the tumor-associated macrophages, suggesting that HO-1 may modulate the tumor microenvironment [73]. In non-small cell lung Cancer (NSCLC), the invasive and migratory abilities promoted by high concentrations of glucose increase with the overexpression of HO-1, while decreasing with its silencing [74]. Since an increase of HO-1 expression is associated with migration and invasiveness, a correlation between HO-1 and the occurrence of EMT, a hallmark of metastatic process, has been investigated. In ovarian cancer, EMT is induced by the inhibition of autophagy and the increase of HO-1 expression and intracellular ROS, whereas the inhibition of HO-1 impairs migration and invasion by inverting EMT [75]. HO-1 may be considered as a novel target for more effective cancer treatments. In this context, it has been demonstrated that pharmacological inhibitors or silencing of HO-1 make tumors more sensitive to therapies, inducing ROS generation and inhibiting tumor growth. Recently, numerous imidazole-based non-porphyrin HO-1 inhibitors were developed. These compounds possess a high selectivity and specificity toward HO-1 and their effective antitumor activity has been demonstrated in vitro and in vivo [76]. On the contrary, notwithstanding in some cases chemo- or radio-therapy contribute to the increase of HO-1 expression, tumor cells expressing high levels of HO-1 are less sensitive to treatment with doxorubicin, cisplatin or gemcitabine in urothelial cancers, cholangiocarcinoma, pancreatic and lung cancer cells [77,78]. For instance, in pancreatic cancer cells the hypoxic conditions contribute to HO-1 up-regulation, but the inhibition of HO-1 using specific inhibitors under hypoxia reduces cell proliferation, and enhances sensitivity to gemcitabine in vitro. Furthermore, the combined treatment with gemcitabine and HO-1 inhibitors in orthotopic models of pancreatic cancer restrains tumor growth and metastasis formation [79].

### 3.3. The Role of Tumor-Associated Macrophages in Iron Metabolism

**Figure 2:**
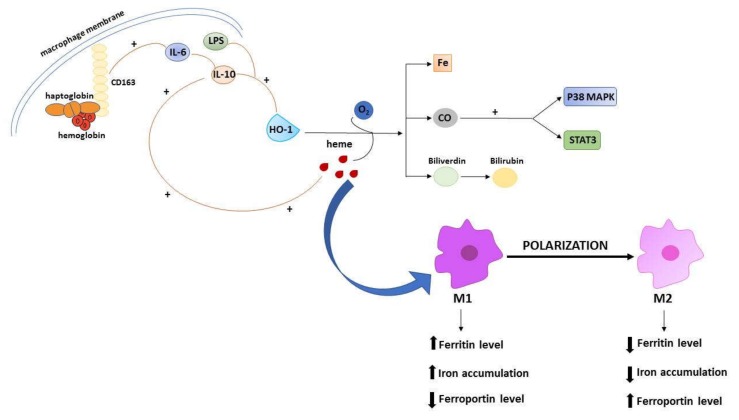
Schematic illustration of the iron-related mechanisms participating in macrophages polarization. The M1 and M2 polarization is accompanied by iron-related phenotypic changes involving ferritin, ferroportin, and iron levels. Macrophages polarization is driven by cytokines activation, such as IL-10 and IL-6. IL-10 induces the activity of HO-1 that oxidizes heme, releasing carbon monoxide (CO), iron and biliverdin. CO activates STAT 3 and p38 MAPK pathways, further promoting the release of IL-10 and the expression of HO-1. IL-6 and IL-10 enhance the expression of CD163 receptor that binds hemoglobin in complex with haptoglobin.

A large amount of evidence points to the relevant involvement of macrophages in the regulation of iron metabolism, due to their reprocessing the iron that is released from damaged erythrocytes, and then returning it to circulation [80,81]. In general, macrophages in the tumor microenvironment (tumor associated macrophages, TAMs) show an iron-releasing phenotype, which has been traditionally framed in the poor prognostic index associated with a high amount of TAMs [82]. However, being plastic elements, macrophages could be polarized into two main subtypes: The classical M1 and alternative M2 macrophages. Noteworthy, the polarization processes across the two opposite/distinct phenotypes include changes in iron homeostasis, defined by modifications of the expression profile of genes implicated in iron metabolism. The switch across M1 and M2 state involves iron-related phenotypic transitions that favor high (M1) versus low (M2) accumulation of intracellular iron. Thus, M1 macrophages exhibit a phenotype with high level of ferritin and low level of ferroportin, while M2 macrophages show low level of ferritin and high level of ferroportin [83]. The expression of iron-related genes in macrophages is promoted by inflammatory cytokines such as IL-6, IL-10 or bacteria-derived lipopolysaccharide (LPS). In murine macrophages, IL-10 induces HO-1 expression through the p38-mitogen activated protein kinase-dependent pathway (MAPK) and contributes to their polarization. Briefly, the activity of HO-1 with IL-10 contributes to macrophages polarization through a positive feedback loop in which the degradation of heme leads to the production of carbon monoxide (CO) that activates p38 MAPK plus the signal transducer and activator of transcription 3 (STAT3) pathway. They further promote the secretion of IL-10, which in turn induces the expression of HO-1 [84]. In addition, IL-6 and IL-10 induce the expression of CD163, a key membrane protein in macrophages, which acts as an endocytic scavenger receptor that binds hemoglobin in complex with the membrane protein haptoglobin (Figure 2). Moreover, IL-6 or LPS induce the expression of hepcidin in human macrophages, determining an iron preservation phenotype. Upon LPS treatment of M1 macrophage, less iron transporter ferroportin coupled with low CD163 and high levels of ferritin are expressed favoring iron preservation. Conversely, M2 macrophages express high levels of ferroportin and CD163, which cooperate in contrasting ferritin, favoring iron release [85]. This feature is related with the function of CD163, and its involvement in pro-tumor switch of macrophages in cancer progression in humans and mice. Shiraishi et al. demonstrated a macrophage-induced tumor cell proliferation in co-cultures of human monocyte-derived macrophages with sarcoma cell lines, which was specifically abolished by silencing CD163. Furthermore, tumors developed from sarcoma cells in CD163-deficient mice were smaller than those in wild-type mice [86]. Macrophages represent the main component of leukocyte infiltrating the tumor microenvironment and influence the formation, growth and metastasis of tumor by interacting with cancer cells. During tumor progression, M1 macrophages are characterized by supporting the immune system that promotes tumor cell removal and are recruit in the tumors. For instance, the polarization of M1 macrophage prevent the progression of glioblastoma [87]. In response to different stimuli, TAMs can switch to cancer-promoting M2 phenotype, and hence support almost all hallmarks of cancer by producing growth factors, and by contributing to extracellular matrix remodeling. TAMs also provide various factors inducing angiogenesis (such as VEGFA), or metabolism regulators, including c-myc and HIF. It has been demonstrated that HIF can induce an alternative activation of macrophage in glioblastoma, while c-myc is highly expressed in TAMs. [88]. Taken together, these findings explain the crucial role of iron metabolism in the polarization of macrophages and their involvement in cancer through the metabolism of this inorganic ion. To the edge of its relevance in macrophage polarization, one interesting aspect deserving investigation is the role possibly exploited by iron with respect to TAMs as treatment target in oncology [89]. In some instance, it has been shown that the density of TAMs at tumor front can predict the responsiveness to chemotherapy. This is the case for colorectal cancer [90], in which the density of CD68+ TAMs interact with chemotherapy in predicting patient outcome, possibly linked to a skew of macrophage polarization toward a M1 anti-tumor role enhanced by 5-fluorouracil, as observed in vitro.

### 3.4. Mini-Summary

In this section, we have underlined the central role of iron in producing ROS and contributing to cell death pathways, such as ferroptosis. Iron-induced free radicals cause oxidative stress and damage to DNA, proteins and cellular organelles, that contribute to the development of cancer. Furthermore, ROS generation induces the activity of the metabolizing enzyme HO-1, involved in the progression of hematologic and solid malignancies. HO-1 expression is detectable also in TAMs, and participates in tuning tumor microenvironment. The studies above-cited support the crucial iron-related mechanisms contributing to macrophage polarization and their supportive role in cancer progression.

## 4. Interplay Between Iron Metabolism and Immune System in Tumor

The role of iron in cancer progression is closely associated with its interferences with the immune response. To control iron availability, macrophages and T cells can modify their phenotype resembling the mechanisms observed in defense intervention against pathogens, mechanisms which may be activated also at the local tumor level. In fact, cancer cells are themselves as well recognized as foreigners, and immune cells modify their polarization also modulating metabolites involved in iron metabolism.

Accordingly, tumor cells compete with immune cells for iron in the microenvironment in which the pro-inflammatory cytokines promote iron sequestration in macrophages, and induce ROS production as anti-tumor defense response [91].

Tumor progression is sustained when cancer cells escape the immune system and/or acquire an immunosuppressive phenotype so that, interacting with tumor-infiltrating immune cells, they promote tumor growth, survival, angiogenesis, and metastasis [92]. As opposed to iron impounding, the anti-inflammatory macrophages and lymphocytes exhibit an iron release phenotype, providing iron in the tumor microenvironment. Therefore, tumor cells are iron users, while lymphocytes and macrophages would act as iron-releasing cells. Moreover, it has been clarified that macrophages are able to secrete ferritin, its expression being found mainly in the stromal compartment in which ferritin promotes tumor growth [93].

The metabolic signature of the microenvironment is responsible for the immunosuppressive nature of tumors. This is related with the polarization of T cells towards a T regulatory phenotype which induces an inability of the cellular immune system to effectively mount a T-cell response and, consequently, an exhaustion of T cell as well as the expression of immune checkpoints such as programmed cell death protein 1 (PD1) [94].

Immunity and metabolism are closely connected. In particular, proteins involved in iron homeostasis may influence lymphocyte populations leading to aberrant ratios of T cells subsets.

For instance, *HFE* protein is at the interface of iron metabolism and immune system linking *HFE* to antigen presentation by major histocompatibility complex class I (MHC-I) molecules [95].

MHC molecules are glycoproteins of the cell surface specialized in presenting antigens to T lymphocytes. This mechanism initiates an immune response for the elimination of infected or damaged cells by recognizing the foreign antigens. Classical MHC-I molecules are associated with cellular adaptive immunity, while non-classical MHC-I molecules promote an alternative form of immune suppression and surveillance that support both innate and adaptive immune response [96]. *HFE* is considered a non-classical MHC-Ib, but it has no ability to bind antigens. Nevertheless, *HFE* is recognized by T cells and is able to modulate the T cell population so that CD4/CD8 ratio is unbalanced in patients with *HFE* mutations [97].

Generally, non-classical MHC-I molecules have been shown to bind natural killer (NK) cells involved in the innate immune response. In contrast, *HFE* is not recognized by NK cells and its expression does not modify the reactivity of NK cells. Until now, anomalies in NK populations have not been described in hereditary hemochromatosis [98]. The numerous immune defects reported in HH patients strongly suggest the relationship between hemochromatosis and modification of the immune response. Iron can influence the phenotype of immune cells and inhibits the surface expression of CD2 and CD4 on T lymphocytes [99]. Several studies on HH are associated with an increase of CD8+ T cell subsets, such as regulatory CD8+/CD28− T cells, which is coupled with a decrease of CD8+/CD28+ T cells and diminished activity of cytotoxic T lymphocytes. Furthermore, the cytokine profiles in HH patients presented high levels of IL-10 and IL-4 produced by CD8+ T cell subset [100]. Recently, some studies have focused on the impact of the presence of wild-type or mutated *HFE* on CD8+ T lymphocytes activation. Wild-type *HFE* inhibits the secretion of T cell cytokines and the expression of lymphocyte activation markers. This inhibition implicates the α-1-2 domains of wild-type *HFE* and is independent of MHC I expression level [101].

Taken together, these findings suggest an important role of wild-type *HFE* in altering the reactivity of CD8+ T lymphocytes, probably modulating the antigen immunogenicity.

### Mini-Summary

We can summarize that immune response and iron metabolism are strongly associated in the context of cancer. The tumor-infiltrating immune cells modify their polarization modulating metabolites involved in iron metabolism and interacting with cancer cells to promote tumor growth.

## 5. Conclusions

Iron is involved, in different ways, at various stages of tumor initiation and progression, and contributes to growth and proliferation of cancer cells. Accumulating evidence implicates alterations of iron metabolism and ensuing excess of iron as crucial features for cancer development. This aberrant accumulation of iron results in ROS generation and oxidative stress which incurs damage to DNA, proteins and other biomolecules. Variants in *HFE* gene are associated with deregulation of iron homeostasis lead to hereditary hemochromatosis and to variably increased risks of cancer. However, the association between *HFE* variants and the development of cancer needs further clarification. Iron is also involved in the modulation of the immune responses, and is seized in macrophages that when showing an iron-releasing phenotype interact with cancer in tumor micro-environment cells to sustain tumor progression.
